# Nucleocapsid protein of SARS‐CoV‐2 is a potential target for developing new generation of vaccine

**DOI:** 10.1002/jcla.24479

**Published:** 2022-05-09

**Authors:** Weixu Feng, Yunru Xiang, Lianpeng Wu, Zhuo Chen, Qingfeng Li, Jun Chen, Yanru Guo, Dandan Xia, Na Chen, Lifang Zhang, Shanli Zhu, Kong‐Nan Zhao

**Affiliations:** ^1^ 26453 School of Basic Medical Science Wenzhou Medical University Wenzhou China; ^2^ Department of Laboratory Medicine The Sixth People Hospital of Wenzhou Wenzhou China; ^3^ Department of Obstetrics and Gynaecology The Second Affiliated Hospital and Yuyin Children Hospital of Wenzhou Medical University Wenzhou China; ^4^ 26453 Australian Institute for Bioengineering and Nanotechnology The University of Queensland St Lucia Queensland Australia

**Keywords:** antibody, immune response, nucleocapsid protein, SARS‐CoV‐2, vaccine

## Abstract

**Background:**

SARS‐CoV‐2 has spread worldwide causing more than 400 million people with virus infections since early 2020. Currently, the existing vaccines targeting the spike glycoprotein (S protein) of SARS‐CoV‐2 are facing great challenge from the infection of SARS‐CoV‐2 virus and its multiple S protein variants. Thus, we need to develop a new generation of vaccines to prevent infection of the SARS‐CoV‐2 variants. Compared with the S protein, the nucleocapsid protein (N protein) of SARS‐CoV‐2 is more conservative and less mutations, which also plays a vital role in viral infection. Therefore, the N protein may have the great potential for developing new vaccines.

**Methods:**

The N protein of SARS‐CoV‐2 was recombinantly expressed and purified in *Escherichia coli*. Western Blot and ELISA assays were used to demonstrate the immunoreactivity of the recombinant N protein with the serum of 22 COVID‐19 patients. We investigated further the response of the specific serum antibodies and cytokine production in BALB/c mice immunized with recombinant N protein by Western Blot and ELISA.

**Results:**

The N protein had good immunoreactivity and the production of IgG antibody against N protein in COVID‐19 patients was tightly correlated with disease severity. Furthermore, the N protein was used to immunize BALB/c mice to have elicited strong immune responses. Not only high levels of IgG antibody, but also cytokine‐IFN‐γ were produced in the N protein‐immunized mice. Importantly, the N protein immunization induced a high level of IgM antibody produced in the mice.

**Conclusion:**

SARS‐CoV‐2 N protein shows a great big bundle of potentiality for developing a new generation of vaccines in fighting infection of SARS‐CoV‐2 and its variants.

## Graphical abstract

The SARS‐CoV‐2 N protein shows a great big bundle of potentiality for developing a new generation of vaccines in fighting SARS‐CoV‐2 infection.

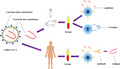

## 1 INTRODUCTION

COVID‐19 is caused by infection with the severe acute respiratory syndrome coronavirus 2 (SARS‐CoV‐2) virus. The SARS‐CoV‐2 virus has swept the world with more than 400 million people getting infection, which has a huge impact on human health and life since 2020.^1^, ^2^ Therefore, vaccines that prevent SARS‐CoV‐2 infection are particularly important. Currently, several types of vaccines against SARS‐CoV‐2 infection have been applied in the world, which include mRNA and DNA vaccines, inactivated vaccines, vector vaccines, etc. These vaccines are mainly aimed at the spike glycoprotein (S protein) of the SARS‐CoV‐2 virus. The S protein is responsible for binding to the receptor (ACE2) on the host cell leading to the virus entry into human body.^3^, ^4^ Thus, the S protein is a preferred target for developing vaccines that block the binding of the S protein to ACE2 to prevent the SARS‐CoV‐2 infection.^5^ However, with the development of the epidemic, high frequencies of mutations of the S protein have made the existing vaccines facing great challenges. It has been found that two mutants (D614G and N501Y) enhance markedly the affinity of the S protein to ACE2 to promote the entry of the SARS‐CoV‐2 into human cells, thereby increasing the viral infectivity.^6^, ^7^ Furthermore, E484K and E484Q mutations can confer advantage to the virus to escape from neutralizing antibody.^8^, ^9^ Since late 2020, Delta variant that swept the world has become the most threatening virus variant.^10^ The Delta variant carried three new mutations: L452R, P681R, and T478K.^11^, ^12^ L452R allows the virus to evade the antibody action by destroying the binding of the anti‐S antibodies to the S protein.^13^ P681R enhances the fusion process between the virus and the receptor cell membrane and T478K augments the binding ability of the S protein to ACE2.^14^, ^15^ The synergistic effects of the three mutations have made Delta variant to be the most pathogenic variant. Nonetheless, there is a need to update the currently existing vaccines against the infection of SARS‐CoV‐2 variants. Therefore, vaccines targeting other viral antigens may be used as a supplement of the existing vaccines to withstand the mutation of SARS‐CoV‐2. Besides the S protein, the structural proteins of the SARS‐CoV‐2 also include membrane protein (M protein), envelope protein (E protein), and nucleocapsid protein (N protein).^16^ The N protein binds to genomic RNA to form a complex, interacting with the viral M protein to improve the efficiency of virus transcription and assembly during the virus assembly process.^17^ The N protein of SARS‐CoV‐2 has 90% amino acid similarity to that of SARS virus while the amino acid similarity of the S proteins between two viruses is only 76%.^18^ The N protein is also more conservative and lower mutation rate compared with the S protein.^19^, ^20^, ^21^, ^22^, ^23^ Furthermore, many coronaviruses have shown that their N proteins are highly immunogenic and are abundantly expressed during infection.^24^ Thus, either N gene or protein has been studied for developing vaccines against the infections of these coronaviruses. Zivcec and colleagues used adenovirus that carried Crimean‐Congo hemorrhagic fever virus (CCHFV) N gene to immunize the IFNAR−/− mice, and then infected the IFNAR−/− mice with CCHFV.^25^ They observed that N gene immunization‐induced antibody could completely protect IFNAR−/− mice from death.^25^ It has been reported that immunization with measles virus N protein led rats to resist infection with measles virus and protect from encephalitis.^26^ Furthermore, immunization of the lymphocytic choriomeningitis virus (LCMV) N protein could prevent mice from infection with LCMV.^27^ Apparently, the N proteins from different viruses used as a vaccine can protect the hosts against the virus infections. Thus, a vaccine that targets SARS‐CoV‐2 N protein might be effective against the infection of this virus and its variants.

Published studies have shown that high levels of anti‐N protein antibody were detected in COVID‐19 patients, indicating that the N protein could stimulate human immune responses to SARS‐CoV‐2 virus.^28^, ^29^ In animals, Ahlen and colleagues have reported that New Zealand rabbits and C57BL/6 mice immunized with either SARS‐COV‐2 N gene expression plasmid or N protein could produce anti‐N antibody of 10^4^–10^5^ titer in rabbits and increase the number of IFN‐γ spot‐forming splenocytes in mice.^30^, ^31^ Furthermore, they observed that intranasally immunization of recombinant adenovirus type 5 carrying the SARS‐CoV‐2 N gene induced CD8+T cell response in the lung and CD4+T cell response in the spleen in BALB/c mice.^32^ In addition, SARS‐CoV‐2 N protein expressed by *E. coli* can be transmitted to the skin of BALB/c mice to induce significant humoral and cellular immunity in mice.^33^ These animal experiments have shown that the N protein has good immunogenicity, which can stimulate strong humoral immunity and cellular immunity, Thus the produced anti‐N protein antibody may play an important role in protecting body against SARS‐CoV‐2 infection. However, the published human patient and animal studies on the immunogenicity of the SARS‐CoV‐2 N protein were separately conducted; none of the results was validated from the reciprocal experiments. Animal experiments sometimes predict well the human reactions, such as the typically historical example that penicillin was observed to protect both mice and humans from staphylococcal infections.^34^

Considering that the N protein was more conservative and lower mutation, which can nicely be applied to fight variants. Therefore, we aimed to study the immunogenicity of the SARS‐CoV‐2 N protein in both human counterparts and animal model to observe that whether N protein possessed potential to serve as an effective vaccine candidate. In this study, we first expressed recombinantly SARS‐CoV‐2 N protein in *E. coli* system after codon‐optimizing the SARS‐CoV‐2 N gene. We then used specific serum antibody from COVID‐19‐infected patients to determine the immunoreactivity of recombinant N protein. We investigated further the response of the specific serum antibodies (IgG and IgM) and cytokine (IFN‐γ) production in BALB/c mice immunized with recombinant N protein. From our reciprocal experiments, we demonstrated that SARS‐CoV‐2 N protein is a powerful antigen for developing a new generation of vaccines.

## 2 MATERIALS AND METHODS

### 2.1 Expression, purification, and identification of the N protein of SARS‐CoV‐2

To express the N protein of SARS‐CoV‐2, we synthesized artificially a DNA sequence of the N protein that was converted from its amino acid sequence. The N gene DNA sequence plus a His‐tag gene sequence was codon‐optimized for a prokaryotic expression system. The codon‐optimized N gene plus a His‐tag gene was inserted into a prokaryotic expression vector pET21a(+) at N*deI* and X*hoI* sites to construct a pET21a(+)/SARS‐CoV‐2‐N expression plasmid. The inserted N gene DNA was sequenced and confirmed to be error free by sequence analysis and restriction endonuclease digestion analysis. The pET21a(+)/SARS‐CoV‐2‐N expression plasmid was transformed to *E. coli* BL21 (DE3), which was induced to express recombinant SARS‐CoV‐2 N protein by treatment of isopropyl‐β‐D‐thiogalactopyranoside (IPTG) at 0.2 mM (Generay Biotech). The recombinant SARS‐CoV‐2 N protein that carried the His tag was purified with Ni‐NTA agarose (QIAGEN). The expressed recombinant SARS‐CoV‐2 N protein was identified by sodium dodecyl sulfate‐polyacrylamide gel electrophoresis (SDS‐PAGE) and Western Blot analysis using HRP‐anti‐His monoclonal antibody (MULTI SCIENCES). Its concentration was determined by the BCA protein quantification method. The purified recombinant SARS‐CoV‐2 N protein was stored at −20℃.

### 2.2 Human serum sample collection

Serum samples were collected from 22 patients recovered from COVID‐19 infection and 10 health participants with written consent. All the serum experiments were carried out in the Clinical Laboratory of the Sixth People's Hospital of Wenzhou. Detection of antibody specific to the N protein of the SARS‐CoV‐2 was carried out by Western Blot and indirect ELISA. All the COVID‐19 cases from the Sixth People's Hospital of Wenzhou were confirmed by clinical diagnosis. The study was approved by the Human Research Ethics Committee of the Sixth People's Hospital of Wenzhou. Collected serum samples were stored at −80℃ for the study.

### 2.3 Animal vaccination

Female BALB/c mice at 6 to 8 weeks old were purchased from Hangzhou Ziyuan Experimental Animal Technology Co. Ltd. All the mice were kept in the animal facility of Wenzhou Medical University (Wenzhou, China). All the animal experiments were performed according to the approved protocols and in accordance with the proposals of use and care of laboratory animals. The BALB/c mice were randomly divided into two groups, with each group having 7 mice. One group of the mice was immunized with purified recombinant N protein together with Freund's adjuvant. At week 0, each mouse was subcutaneously injected with 400 μl of the mixture of N protein (50 μg/200 μl) and complete Freund's adjuvant (200 μl) (Sigma). At week 2 and 4, each of the mixture of N protein/complete Freund's adjuvant‐immunized mice was further immunized with N protein (50 μg/200 μl) and incomplete Freund's adjuvant (200 μl). The other group of mice was only subcutaneously immunized with a mixture of PBS/Freund's adjuvant as a negative control. At week 0, each mouse was subcutaneously injected with 400 μl of the mixture of PBS (200 μl) and complete Freund's adjuvant (200 μl). At week 2 and 4, each of the control mice was further injected 200 μl of PBS and 200 μl of incomplete Freund's adjuvant. Tail veins of the immunized mice were used for collecting venous blood samples at week 0 (W0), week 1 (W1, one week after first immunization), week 3 (W3, one week after second immunization), week 5 (W5, one week after third immunization), and week 7 (W7, three weeks after the third immunization). The mice were sacrificed after the last blood collection. The serum samples collected from the mice bloods were stored at −80℃ for subsequent experiments.

### 2.4 Detection of IgG antibody against N protein in COVID‐19 patient's serum

The immunoreactivity of the N protein was detected by Western Blot and indirect ELISA.

#### 2.4.1 Western Blot analysis

12% SDS‐PAGE was used for electrophoreses and Western Blot. The PBS buffer and irrelevant protein (cellular protein) were used as controls. The serum at 1:100 dilution from COVID‐19 patients used as primary antibody was incubated at 37℃ for 2 h, then washed with TBST 3 times and 5 min each time. HRP‐conjugated Goat anti‐human IgG(H + L) (Abcam) at 1:50,000 dilution was used as the secondary antibody that was incubated at 37℃ for 2 h, then the TBST washed 5 times and 5 min each time. Finally, Clarity western ECL substrate (Bio‐Rad) was used to visualize the band.

#### 2.4.2 Indirect ELISA

96‐wells plate was coated with 1 μg/well of the purified N protein and incubated at 4℃ overnight. Then, the N protein‐coated plate was blocked with 5% skimmed milk at 4℃ overnight. After that, the plate was washed with PBST 3 times. 100 µl of patient serum at 1:100 dilution were added to the N protein‐coated plate and incubated at 37℃ for 2 h. Then, the patients‐serum incubated plate was washed with PBST 5 times. HRP‐goat anti‐human IgG(H + L) used as the secondary antibody with dilution of 1:50,000 dilution was added to the plate and incubated at 37℃ for 2 h. Then, the secondary antibody‐incubated plate was washed with PBST 5 times. After washing, 100 μl of the chromogenic substrate TMB was added for reaction for 5 min under dark condition. The reaction was stopped by adding 100 μl of stop solution. The absorbance (OD) at 450 nm was measured by the Bio‐Tek ELISA microplate reader. Serum of health participants and PBS buffer were used as negative controls. All the samples for the indirect ELISA were performed in triplicate, and the antibody titer in the patient serum was additionally detected.

### 2.5 Detection of specific antibody in sera of the N protein‐immunized mice

Specific antibody generation in the mice immunized with the N protein was determined by Western Blot and indirect ELISA.

#### 2.5.1 Western Blot analysis

At week 5, serum at 1:10,000 dilution from the N protein‐immunized mice was used as primary antibody, HRP‐conjugated Goat anti‐mouse IgG(H + L) (MULTI SCIENCES) at 1:50,000 dilution was used as the secondary antibody. Clarity Western ECL substrate (Bio‐Rad) was used to visualize the band. In addition, serum collected from the N protein‐immunized mice at week 7 at 1:100 dilution was used to determine two main types of antibodies IgG and IgM. Both HRP‐goat anti‐mouse IgG(H + L) (MULTI SCIENCES) at 1:50,000 dilution and HRP‐goat anti‐mouse IgM (Bioss) at 1:5000 dilution were used as the secondary antibodies. The other procedures were the same as that for the Western Blot assay.

#### 2.5.2 Indirect ELISA

A 96‐wells plate was coated with purified N protein (1 μg/well) and incubated at 4℃ overnight. Serum samples from the mice immunized with either N protein or PBS (negative control) at the dilution of 1:100–1:10,000,000 dilution were added to the plate. HRP‐goat anti‐mouse IgG(H + L) (MULTI SCIENCES,) diluted to 1:50,000 dilution was used as the secondary antibody for detection. Furthermore, serum collected from the N protein‐immunized mice at week 7 at 1:100 dilution was used for quantifying IgG and IgM. HRP‐goat anti‐mouse IgG(H+L) (MULTI SCIENCES) and HRP‐goat anti‐mouse IgM (Bioss) were used as above with the same dilution for the detection. The other procedure was the same as that for the ELISA assay.

### 2.6 Detection of serum IFN‐γ in immunized mice

IFN‐γ production in serum collected from the N protein‐immunized mice at week 7 post‐immunization was determined by double‐antibody sandwich ELISA. The specific anti‐mouse IFN‐γ antibody was pre‐coated on a high‐affinity ELISA microtiter plate (MULTI SCIENCES). 10 μl of mouse serum diluted in 90 μl of assay buffer were added to each well in a 96‐well plate. The serum‐contained plate was incubated at 37°C for 1.5 h. The plate was then washed by PBST six times. After that, 100 μl of detection antibody (1:100 dilution, Biotinylated antibody) was added to each well. The plate incubated at 37°C for 30 min was washed with washing solution six times. Next, 100 μl of secondary horseradish peroxidase‐labeled streptavidin antibody (Streptavidin‐HRP) at 1:100 dilution was added and incubated at 37°C for 30 min. After washing three times, 100 μl of signal enhancer reagent (1:100 dilution) was added for incubation at 37°C for 15 min. After washing, 100 μl of Streptavidin‐HRP (1:1000 dilution) was again added for further incubation at 37°C for 15 min. After washing, 100 μl of the chromogenic substrate TMB was added for reaction for 10 min under dark condition. The reaction was stopped by adding 100 μl of stop solution. The absorbance (OD) at 450 nm was measured by the Bio‐Tek ELISA microplate reader.

### 2.7 Statistical analysis

One‐way analysis of variance (ANOVA) and independent samples t test were used to determine the differences between the experimental group and the control group. *p* Value less than 0.05 was considered to be statistically significant. All statistical analyses were performed using SPSS software (version 25.0).

## 3 RESULTS

### 3.1 Expression, purification, and identification of the N protein of SARS‐CoV‐2

**FIGURE 1:**
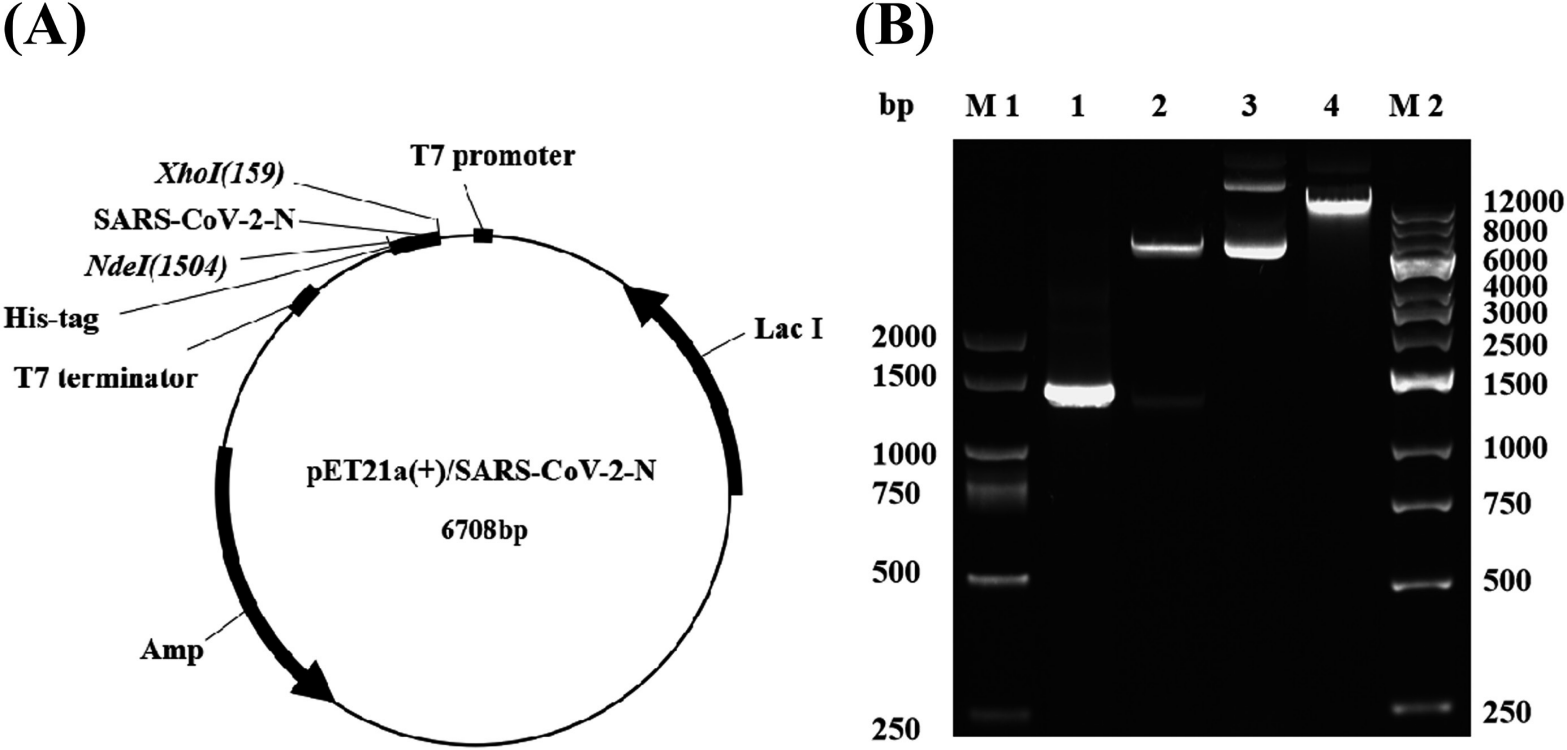
Construction and enzymatic restriction analysis of pET21a(+)/SARS‐CoV‐2‐N plasmid. (A) Schematic structure of pET21a(+)/SARS‐CoV‐2‐N recombinant plasmid. (B) Enzymatic restriction analysis of the constructed pET21a(+)/SARS‐CoV‐2‐N recombinant plasmid. Codon optimized N gene was cloned into the NdeI and XhoI sites of pET21a(+), the recombinant plasmid was digested by restriction enzymes (NdeI and XhoI). M1: DL2 kb DNA marker, M2: 1 kb DNA marker, 1: PCR product of SARS‐CoV‐2‐N gene, 2: pET21a(+)/SARS‐CoV‐2‐N with NdeI/XhoI cut, 3: pET21a(+)/SARS‐CoV‐2‐N, 4: pET21a(+)

**FIGURE 2:**
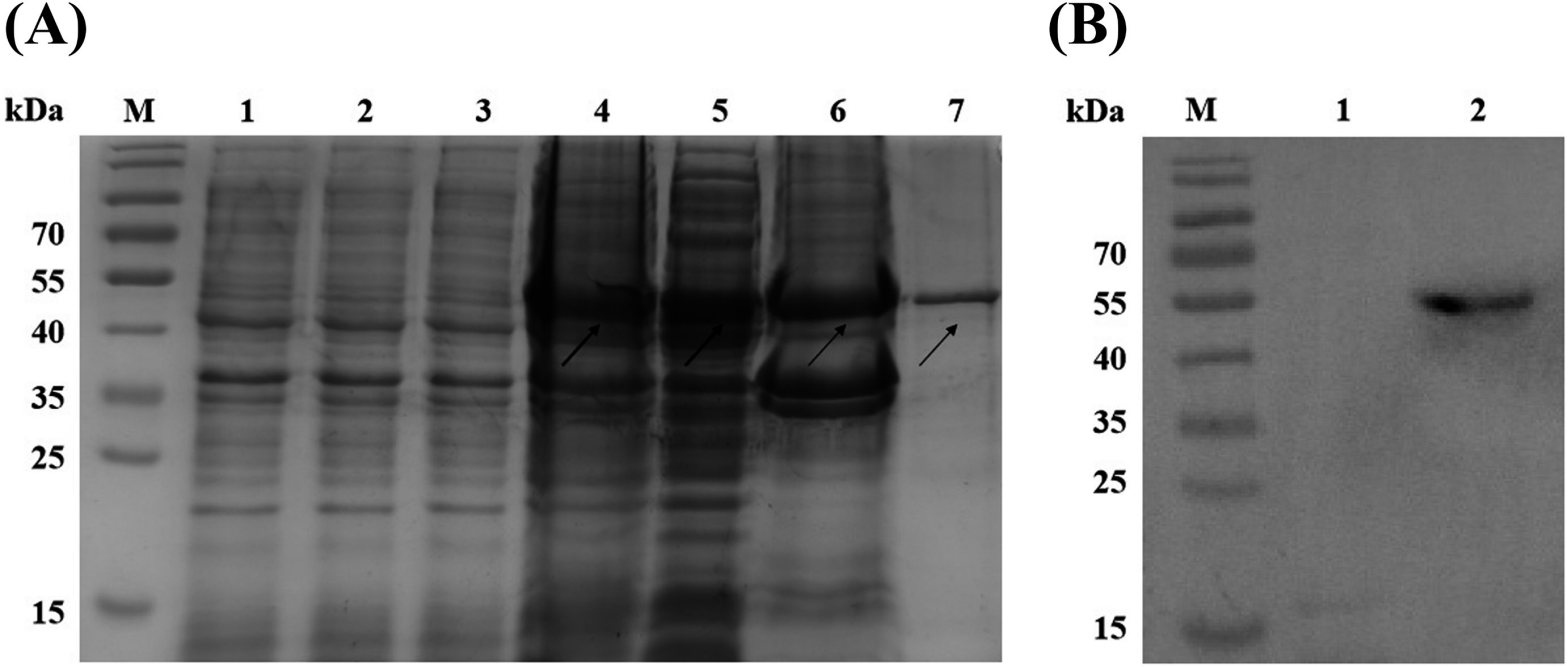
Expression and identification of SARS‐CoV‐2 N protein in *E. coli*. (A) N protein expression was analyzed by SDS‐PAGE. M: Protein marker (kDa); 1: Lysate of *E. coli* BL21, 2: Lysate of pET21a(+) basal plasmid transformed *E. coli* BL21, 3: Lysate of pET21a(+)/SARS‐CoV‐2‐N plasmid transformed *E. coli* BL21 before IPTG treatment, 4: Lysate of pET21a(+)/SARS‐CoV‐2‐N plasmid transformed *E. coli* BL21 after IPTG treatment, 5: Soluble protein, 6: Inclusion body protein, 7: Purified N protein. (B) Identification of purified N protein by Western Blot with anti‐His‐tag monoclonal antibody. M: Protein marker, 1: Lysate of *E*. *coli* BL21, 2: Purified N protein

We cloned a synthesized N gene of SARS‐CoV‐2 with the correct sequence into the prokaryotic expression vector pET21a(+) to construct a pET21a(+)/SARS‐CoV‐2‐N gene expression plasmid (Figure 1A), which was verified by restriction endonuclease digestion analysis (Figure 1B). The pET21a(+)/SARS‐CoV‐2‐N plasmid was transformed to *E. coli* BL21(DE3) with IPTG treatment to induce expression of the N protein identified by SDS‐PAGE. Coomassie staining of the SDS‐PAGE showed that the N protein from the pET21a(+)/SARS‐CoV‐2‐N plasmid was only expressed in *E. coli* BL21(DE3) under the condition of IPTG treatment, which was purified by NI‐NTA agarose affinity chromatography (Figure 2A). The purified N protein was detected by an anti‐His tag monoclonal antibody (Figure 2B). The predicted molecular weight of the N protein was approximately 49 kDa.

### 3.2 Detection of IgG antibody against the N protein in sera of COVID‐19 patients

**FIGURE 3:**
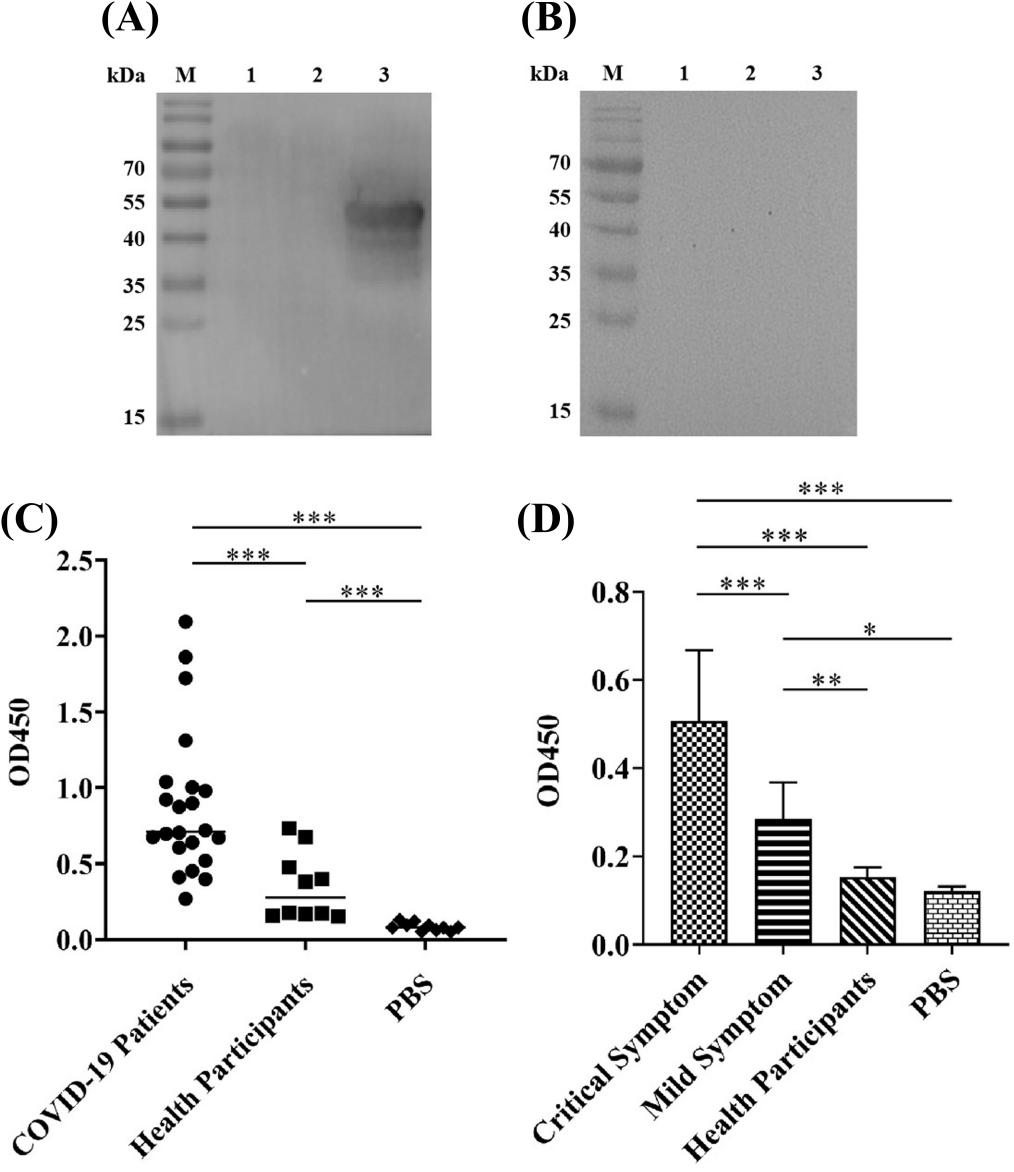
Immunoreactivity analysis of the SARS‐CoV‐2 N protein with COVID‐19 patients serum. Serum samples collected from both COVID‐19 patients and health participants were analyzed by Western Blot and indirect ELISA. Western Blot showed that (A) COVID‐19 patient serum was able to recognize the N protein generating a strong protein band approximately at 49 kDa, implying that N protein‐specific antibody was produced in patients infected with SARS‐CoV‐2 virus and (B) Health participant serum was unable to recognize the N protein without the 49 kDa protein band detected, indicating no N protein‐specific antibody induced in health participant. In figures: M: Protein marker, 1: PBS, 2: 20 µg of irrelevant protein, 3: 20 µg of N protein. Indirect ELISA quantitatively revealed that (C) N protein had strong immunoreactivity and (D) N protein antibody production strength associated with disease servility in COVID‐19 patients. The data were the mean ± SD (n = 3). The experimental details have been described in Materials and Methods Section

Immunoreactivity is the capacity that an antigen is able to specifically bind to an antibody that is induced by itself. A protein has good immunoreactivity, which possesses a stable chemical structure, thereby not easy to be destroyed and then staying longer in the body. This means that this protein can stimulate the body to produce an immune response, because this protein has ample opportunities to contact cells that could produce antibodies.^35^ Thus, we aimed to investigate the immunoreactivity of the N protein with the N protein IgG antibody in COVID‐19 patients' serum. Western Blot results first showed that only the serum from the COVID‐19 patients recognized N protein (Figure 3A), not from the health participants (Figure 3B), suggesting that specific anti‐N protein IgG antibody was induced and the N protein had the specific immunoreactivity. We then used the serum samples collected from both 22 patients and 10 health participants to confirm the immunoreactivity of the N protein by indirect ELISA (Figure 3C). The average OD_450_ value in 22 COVID‐19 patients' serum was 0.88 ± 0.47, which was 2.5 folds higher than that of health participants (0.35 ± 0.21) (*p* < 0.001) (Figure 3C). Results also revealed that the indirect ELISA OD value of the specific anti‐N protein antibody in serum from 5 critically ill COVID‐19 patients was 0.51 ± 0.15, which was significantly higher than that from 4 mild COVID‐19 patients (0.28 ± 0.08, *p* < 0.001), while 5 health participants only had the 0.15 ± 0.03 of OD value, significantly lower than those from both critical and mild COVID‐19 patients (*p* < 0.001) (Figure 3D). The results suggested two points (1). The N protein exhibited specific immunoreactivity and (2). High level of the N protein antibody production in COVID‐19 patients is strongly associated with disease severity.

### 3.3 Antibody production in the N protein‐immunized mice

**FIGURE 4:**
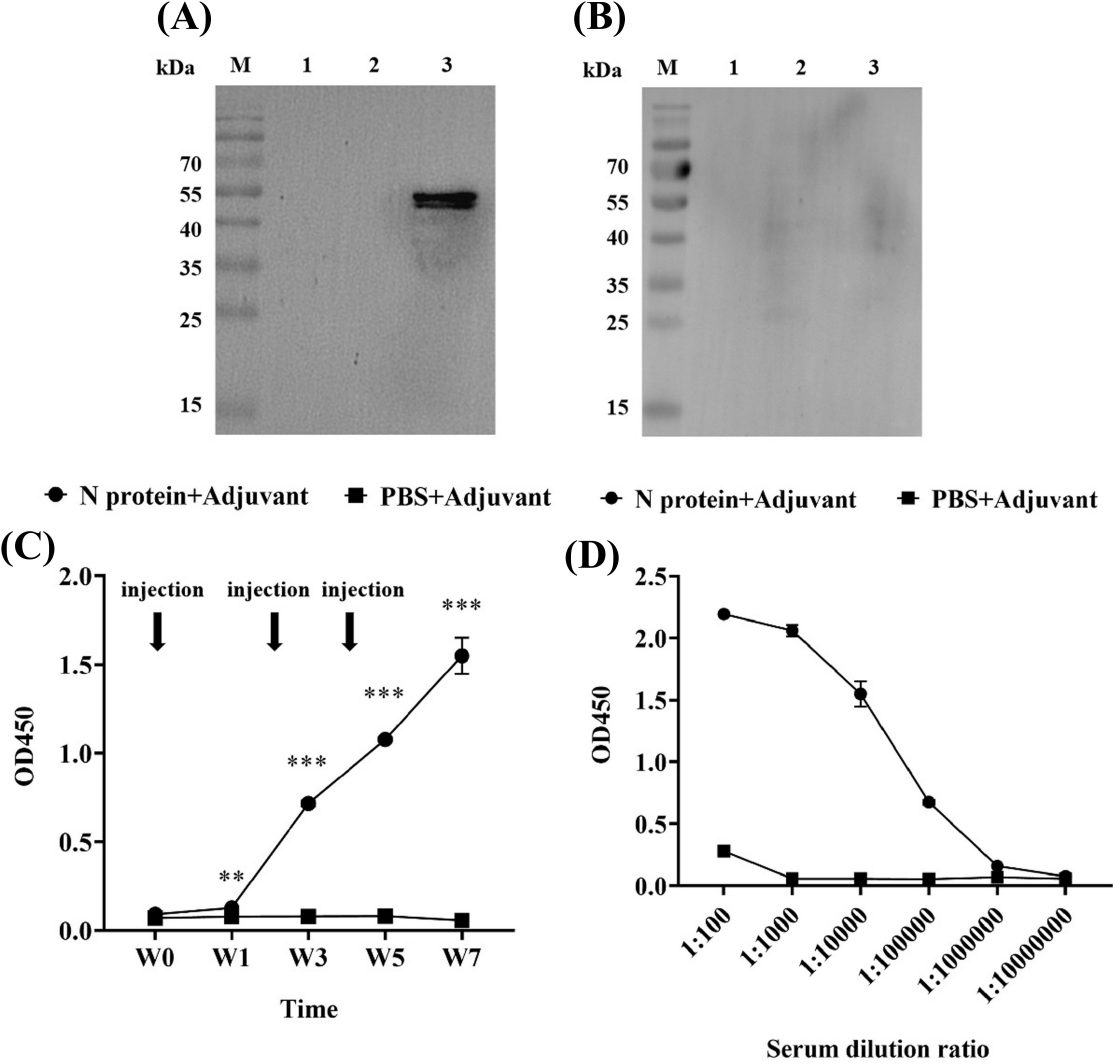
Analysis of specific IgG antibody in the serum of the N protein‐immunized mice. (A) Western Blot showed that serum from the N protein‐immunized mice could specifically recognize the N protein suggesting the production of specific IgG antibody. (B) Serum from the PBS/adjuvant immunized mice failed to recognize N protein indicating no specific IgG antibody production. (C) ELISA quantified the levels of specific IgG antibody in the serum at 1:10,000 dilution collected from the immunized mice at week 0, 1, 3, 5, and 7 post‐immunization, with high level of the specific IgG antibody production detected in N protein‐immunized mice at week 1 post‐immunization. (D) Specific IgG antibody titration analysis revealed that the antibody titer in the serum from the N protein‐immunized mice at week 7 post‐immunization was high up to 1:10,000 dilution

It is well known that the acquired immunity uses specific antigens to mount a humoral immune response by producing five major types of antibodies in which IgG antibody found in all body fluids is the most common antibody (75% to 80%) of all the antibodies and plays a key role in fighting bacterial and viral infections.^36^ Thus, we investigated the production of the N protein specific IgG antibody in sera collected from the immunized mice. Western Blot showed that the N protein was specifically recognized by IgG from the serum of the N protein‐immunized mice (Figure 4A). In contrast, the serum from the mice immunized with a mixture of PBS/adjuvant failed to recognize the N protein, indicating that no humoral immune response was induced in these mice (Figure 4B). Furthermore, the IgG‐specific antibody could be detected in the serum of the mice immunized with the N protein at week 1 post‐immunization while it was undetectable in serum from the mice immunized with a mixture of PBS/adjuvant (*p* < 0.01) (Figure 4C). The highest level of IgG‐specific antibody production in the N protein‐immunized mice was obtained at week 7 post‐immunization, with an OD value up to 1.55 ± 0.10 (Figure 4C). This titer value of the IgG antibody in the 7th‐week serum was obtained based on the titration assay with 1:10,000 dilution (Figure 4D).

**FIGURE 5:**
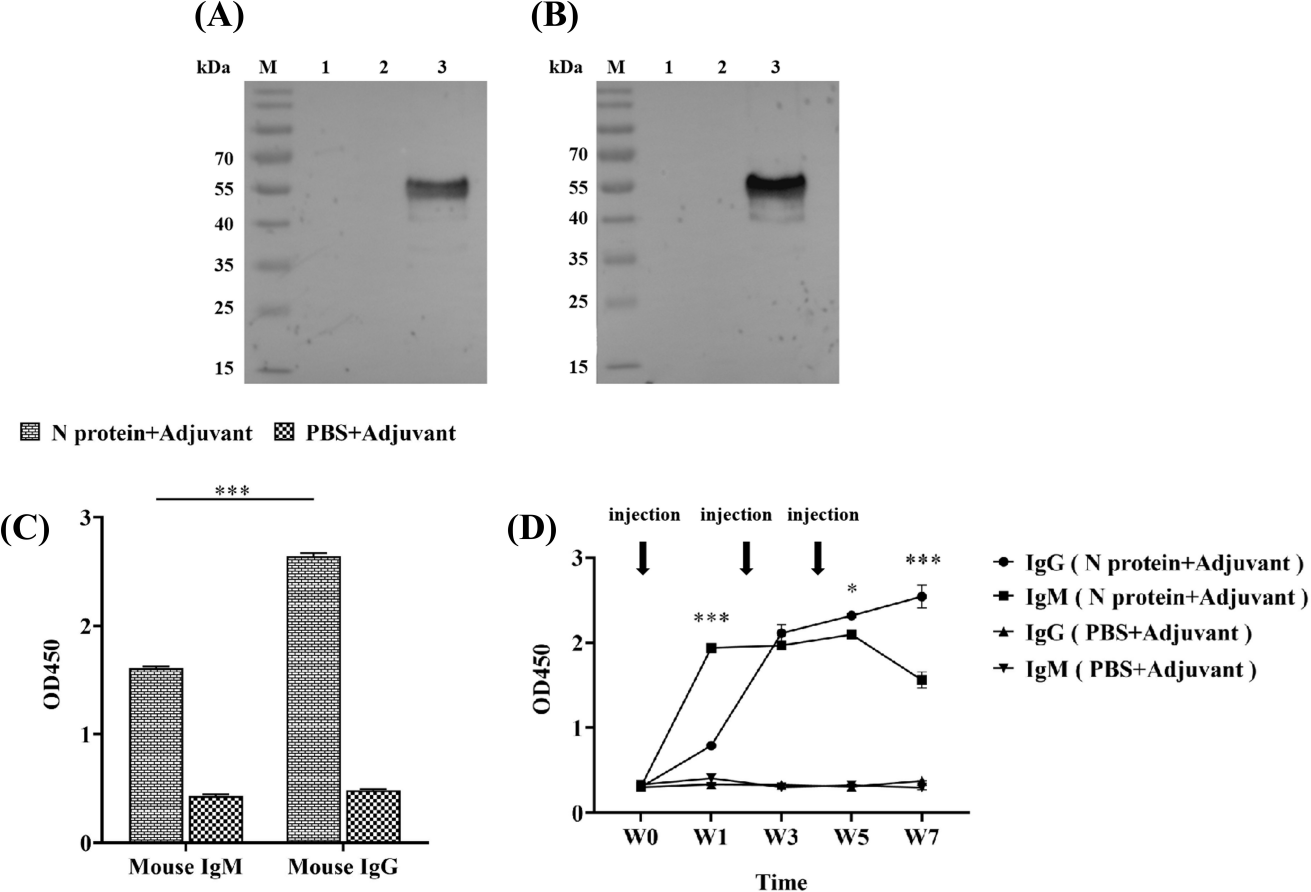
Analysis of specific IgM and IgG antibodies in serum of the N protein‐immunized mice at week 7 post‐immunization. (A) Western Blot showed IgM antibodies detected with strong signals. (B) IgG antibodies detected with strong signals. (C) Quantitative ELISA analysis showed the level of IgG antibody production significantly higher than that of IgM antibody (*p* < 0.001). The data were the mean ± SD (n = 3). (D) Longitudinally quantitative ELISA measurements of both anti‐N protein IgM and IgG antibodies in serum at 1:100 dilution of the N‐protein‐immunized mice from week 0 to week 7 post‐immunization

Considering that IgM antibodies are produced early in the humoral immune response against specific antigen,^37^ we investigated whether specific IgM antibody could be produced in the 7th‐week serum of the N protein‐immunized mice in which the highest level of IgG antibody was yielded (Figure 4C). Western Blot revealed that, similar to the IgG antibody (Figure 5B), the IgM antibody with a strong signal was produced (Figure 5A) in the N protein‐immunized mice at week 7 post‐immunization. Indirect ELISA showed further that the OD value of anti‐N protein IgM antibody was 1.61 ± 0.01 and the anti‐N protein IgG antibody had an OD value of 2.64 ± 0.03, which were 4–5 times higher than those in the control group (IgM, 0.43 ± 0.02 and IgG, 0.48 ± 0.01) (Figure 5C). However, the results showed that production of the IgG antibody was significantly higher than that of the IgM antibody (*p* < 0.001) (Figure 5C) although the IgM is the first antibody produced in the body to fight a new infection with bacterium or virus. Thus, we used ELISA to longitudinally measure the levels of both anti‐N protein IgM and IgG antibodies in the serum of the N‐protein‐immunized mice from week 0 to week 7 post‐immunization (Figure 5D). As shown in Figure 5D, the OD value of the IgM antibody was 1.94 ± 0.34 significantly higher than that of the IgG antibody with an OD value of 0.79 ± 0.02 at week 1 post‐immunization. At week 3, the level of IgM remained constant while the level of IgG has risen to the level of IgM. The results suggested that the IgM antibody with the highest level at week 1 post‐immunization was produced to provide fast protective immunity.

### 3.4 IFN‐γ production in the N protein‐immunized mice

**FIGURE 6:**
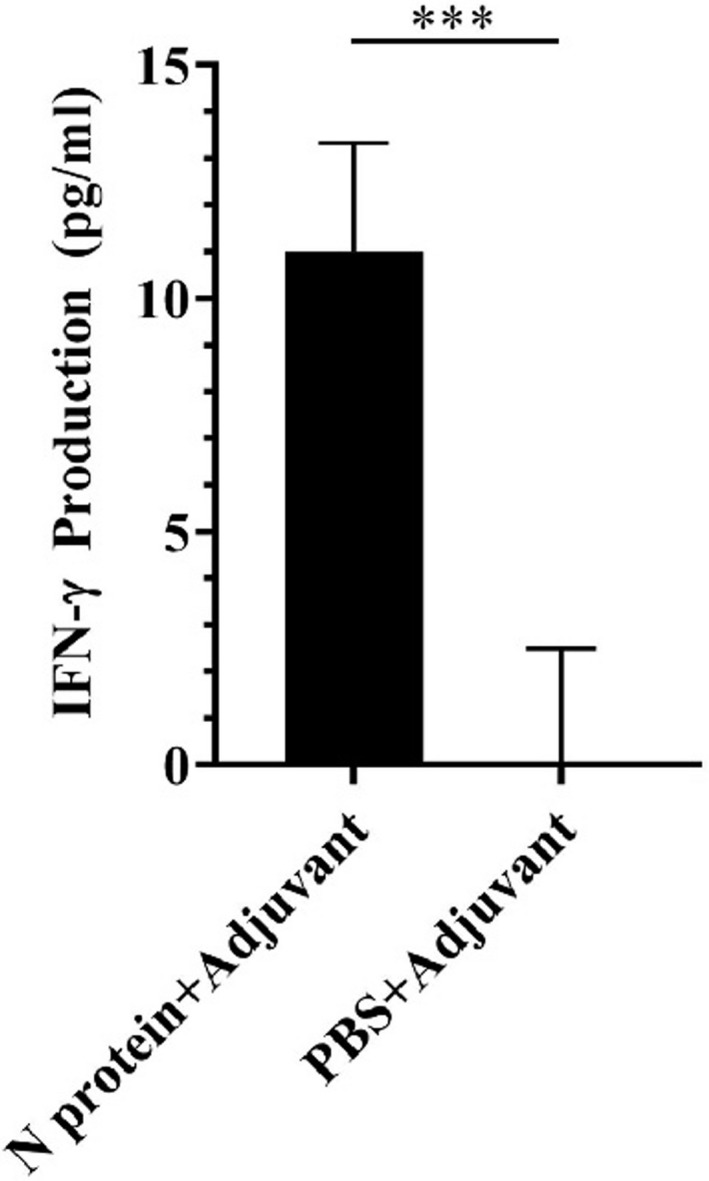
Analysis of IFN‐γ in mouse serum at week 7 post‐immunization. ELISA analysis showed high level of IFN‐γ produced in the N protein‐immunized mice but not in the PBS immunized mice (*p* < 0.001). The data were the mean ± SD (n = 3)

Considering that interferon gamma (IFN‐γ) is a cytokine critical to both innate and adaptive immunity, we investigated whether IFN‐γ was generated in the serum collected from the N protein‐immunized mice at week 7 post‐immunization. Results showed that mice immunized with the N protein could effectively elicit secretion of IFN‐γ in serum (Figure 6). The yield of IFN‐γ in the serum of the N protein‐immunized mice was very high, up to 11.00 ± 2.33 pg/ml (Figure 6), while it was scarcely detected in the serum of the PBS‐immunized mice (Figure 6). The data, together with the production of both IgG and IgM antibodies, suggested that the N protein is highly immunogenic, which could elicit a strong adaptive immune response against SARS‐CoV‐2 infection.

## 4 DISCUSSION

In SARS‐CoV‐2 infection, the S protein plays an important role in viral attachment, fusion, and entry into host cells, which is the first step of viral infection. That was why most of the published studies have focused on targeting the S protein to develop vaccines and therapeutic interventions against the SARS‐CoV‐2 infection.^38^ However, frequent mutations of the SARS‐CoV‐2 S protein at multiple sites make a significant decrease of the protective effect of the developed vaccines leading to COVID‐19 pandemic getting worse although effective neutralizing antibody against the S protein mutants was once observed.^39^ Thus, the developed vaccines or drugs targeting the S protein cannot maintain the long‐term protection from the infection with SARS‐CoV‐2 virus. Once a new mutation occurs, the protective efficacy of the existing vaccines will be markedly declined, and the new variant may have a high probability of causing severe infection. Thus, we need to consider to develop a new generation of the vaccines targeting one of the other three structural proteins of the SARS‐CoV‐2 such as N protein to prevent the infection of SARS‐CoV‐2 variants. Compared with the S protein, the N protein is more conservative and less mutation, and its antibody is more conducive to fight the mutant strains.^23^, ^40^ In addition, the N protein of SARS‐CoV‐2 also plays an equally important role in viral infection. It can enter host cells to promote replication of the viral RNA and accelerate assembly and release of the virus.^41^ Nonetheless, destruction of the SARS‐CoV‐2 N protein may inhibit the viral RNA replication and block the virus transmission in the body, which may solve the problem of the S protein mutations against the infection of SARS‐CoV‐2 virus and its variants.

The N protein of SARS‐CoV‐2 can stimulate human body to produce two types of adaptive immune responses: humoral immunity and cell‐mediated immunity, which enable the human body to defend itself against the virus infection.^33^ Published studies have shown that anti‐N protein antibody can be detected in the patient's serum, which was found to be the earliest and most easily detected antibody.^42^, ^43^ Here, we have verified that the N protein exhibited good immunoreactivity by the serum antibody from the COVID‐19 patients, suggesting that the antibody from COVID‐19 patients might bind to the N protein of the native virus, thereby blocking virus replication and transmission in vivo. In addition, we provided evidence that the level of anti‐N protein antibody in severe COVID‐19 patients was significantly higher than that in mild COVID‐19 patients. Consistent with the previous studies, the results indicated that the activation degree of humoral immunity and cellular immunity in patients infected with COVID‐19 virus was positively correlated with the severity of the disease.^44^ As the aggravation of disease, the virus in the lower respiratory tract of patients replicated actively, it took long time for the virus to be cleared, which depended on the antibody production and cellular immunity.^45^ The production of the antibody may be used for predicting the diagnosis, prognosis, and treatment of the COVID‐19 patients.

Previously published studies have indicated that the N protein from other coronaviruses such as elk coronavirus and infectious bronchitis virus (IBV) also can induce the production of antibody in immunized mice and chickens.^46^, ^47^ Recently, several research groups have used SARS‐CoV‐2 N protein expression plasmid or recombinant SARS‐CoV‐2 N protein to immunize animals.^28^, ^29^, ^30^, ^31^ Immunization of both N protein expression plasmid and recombinant N protein could induce production of IgG antibody and cytokines such as IFN‐γ.^28^, ^29^, ^30^, ^31^ Supporting the previous findings, we here demonstrated further that recombinant N protein immunization could induce to produce high levels of IgG antibody and IFN‐γ in mice. In addition, we for the first time demonstrated the production of the IgM antibody with high titers in the N protein‐immunized mice, revealing that the immune system was actively producing antibodies to elicit the immune responses against the SARS‐CoV‐2 N protein. Both IgM and IgG antibodies are very important in fighting viral infections. The IgM antibody occurs mainly in the early stage, which is the first antibody that the body makes to fight a new infection while IgG appears in the late stage of the viral infection, lasts longer and can provide long‐term protection for the body. Furthermore, IFN‐γ is a key factor in initiating both innate and adaptive immunity by stimulating natural killer cells and neutrophils, which also functions as the primary activator of macrophages. It has been reported that IFN‐γ or its receptor‐deficient mice were very vulnerable to infectious diseases.^48^, ^49^ Immunization of DNA vaccine encoding SARS N protein induced significantly the IFN‐γ production in the mice against the viral infection.^50^

In conclusion, we demonstrated here that (1). the SARS‐CoV‐2 N protein had high immunoreactivity and (2). high level of anti‐N protein antibody produced in COVID‐19 patients was tightly associated with the disease severity. We then proved that immunization of the N protein‐induced strong immune responses to produce not only IgG antibody, but also IgM antibody and IFN‐γ in the immunized mice. The data suggested that the SARS‐CoV‐2 N protein has strong immunogenicity to elicit both humoral immunity and cell‐mediated immunity. Thus, the SARS‐CoV‐2 N protein has the great potential for developing a new generation of vaccines to prevent infection and transmission of SARS‐CoV‐2 virus and its variants.

## CONFLICT OF INTEREST

The authors have declared that no competing interest exists.

## Data Availability

The datasets used and analyzed during the current study are available from the corresponding author.
